# LCL161 increases paclitaxel-induced apoptosis by degrading cIAP1 and cIAP2 in NSCLC

**DOI:** 10.1186/s13046-016-0435-7

**Published:** 2016-09-30

**Authors:** Chengcheng Yang, Huangzhen Wang, Boxiang Zhang, Yimeng Chen, Yamin Zhang, Xin Sun, Guodong Xiao, Kejun Nan, Hong Ren, Sida Qin

**Affiliations:** 1 Department Two of Thoracic Surgery, The First Affiliated Hospital of Xi’an Jiaotong University, 277 West Yanta Road, Xi’an, Shaanxi 710061 China; 2 Department of Oncology, The First Affiliated Hospital of Xi’an Jiaotong University, 277 West Yanta Road, Xi’an, Shaanxi 710061 China; 3 Department of Surgical Oncology, Baoji Central Hospital, Baoji, Shaanxi 721008 China

**Keywords:** NSCLC, cIAP, LCL161, Paclitaxel, Apoptosis

## Abstract

**Background:**

LCL161, a novel Smac mimetic, is known to have anti-tumor activity and improve chemosensitivity in various cancers. However, the function and mechanisms of the combination of LCL161 and paclitaxel in non-small cell lung cancer (NSCLC) remain unknown.

**Methods:**

Cellular inhibitor of apoptotic protein 1 and 2 (cIAP1&2) expression in NSCLC tissues and adjacent non-tumor tissues were assessed by immunohistochemistry. The correlations between cIAP1&2 expression and clinicopathological characteristics, prognosis were analyzed. Cell viability and apoptosis were measured by MTT assays and Flow cytometry. Western blot and co-immunoprecipitation assay were performed to measure the protein expression and interaction in NF-kB pathway. siRNA-mediated gene silencing and caspases activity assays were applied to demonstrate the role and mechanisms of cIAP1&2 and RIP1 in lung cancer cell apoptosis. Mouse xenograft NSCLC models were used in vivo to determine the therapeutic efficacy of LCL161 alone or in combination with paclitaxel.

**Results:**

The expression of cIAP1 and cIAP2 in Non-small cell lung cancer (NSCLC) tumors was significantly higher than that in adjacent normal tissues. cIAP1 was highly expressed in patients with late TNM stage NSCLC and a poor prognosis. Positivity for both cIAP1 and cIAP2 was an independent prognostic factor that indicated a poorer prognosis in NSCLC patients. LCL161, an IAP inhibitor, cooperated with paclitaxel to reduce cell viability and induce apoptosis in NSCLC cells. Molecular studies revealed that paclitaxel increased TNFα expression, thereby leading to the recruitment of various factors and the formation of the TRADD-TRAF2-RIP1-cIAP complex. LCL161 degraded cIAP1&2 and released RIP1 from the complex. Subsequently, RIP1 was stabilized and bound to caspase-8 and FADD, thereby forming the caspase-8/RIP1/FADD complex, which activated caspase-8, caspase-3 and ultimately lead to apoptosis. In nude mouse xenograft experiments, the combination of LCL161 and paclitaxel degraded cIAP1,2, activated caspase-3 and inhibited tumor growth with few toxic effects.

**Conclusion:**

Thus, LCL161 could be a useful agent for the treatment of NSCLC in combination with paclitaxel.

## Background

Lung cancer is the leading cause of death for cancer patients in both males and females worldwide [1]. Non-small cell lung cancer (NSCLC) accounts for more than 80 % of all lung cancers [2], and most patients are diagnosed at an advanced stage without the opportunity for surgery. Paclitaxel, as a first-line chemotherapeutic drug, has been shown to be effective in NSCLC treatment [3]. However, its curative effect is limited, and its side effects are severe. Therefore, novel drug combinations with paclitaxel need to be explored to improve its curative effect and reduce its side effects.

Inhibitor of apoptosis proteins (IAPs) play an important role in cell apoptosis, and they are widely expressed in human tumor tissues [4]. cIAP1 and cIAP2, members of the IAP family, indirectly regulate apoptosis by preventing Smac for inhibiting XIAP-caspase interaction and by preventing the formation of caspase-8-activating platform. Besides, they have a really interesting new gene (RING) domain in their C terminus that promotes self-ubiquitination and ubiquitination of cIAP1 partners such as RIP1, promoting the formation of signaling complex leading to NF-kB activation [5, 6]. Genetic evidence has shown that 11q21-23 (which encodes cIAP1 and cIAP2) is a potential proto-oncogene, and the high expression of cIAP1 and cIAP2 has been closely related to chemoresistance and poor prognosis [7]. Consequently, cIAP1 and cIAP2 are likely to be therapeutic targets with promising potential.

Second mitochondrial-derived activator of caspases (Smac) is a significant endogenous antagonist of IAPs [8]. Smac mimetics are artificially synthesized small-molecule compounds that mimic the apoptotic function of Smac [5]. Previous studies have shown that Smac mimetics induce cell apoptosis by activating caspases [9, 10]. In the past few years, an increasing number of studies have suggested that the function of Smac mimetics in inducing cell apoptosis is inseparably associated with the nuclear factor-kappaB (NF-kB) pathway [11]. For example, the Smac mimetic AEG40730 releases NF-kB inducing kinase (NIK) and activates the non-canonical NF-kB pathway through inducing cIAP autoubiquitination, which causes tumour necrosis factor-α (TNF-α) autocrine signaling and activates TNFR1, leading to apoptosis [12]; BV-6, another Smac mimetic, restrains the canonical NF-kB pathway by degrading cIAP, stabilizing receptor-interacting protein 1 (RIP1) and forming the RIP1-FADD-caspase 8 complex, ultimately activating the death receptor apoptotic pathway [13]. In lung cancer treatment, the sensitivity of NSCLC cell lines to Smac mimetic, Compound 3/4, alone is related to autocrine-secretion of TNFα and the formation of RIP1-dependent caspase-8-activating complex [14]. However, Smac mimetic, JP1201, could sensitize nonresponsive NSCLC cell lines to standard chemotherapy independently of TNF-α secretion [15].

LCL161, a novel Smac mimetic, has been tested as an anticancer agent in phase I and phase II clinical trials. It possesses many advantages such as good tolerability, low toxicity and oral availability [16]. Recently, the anti-proliferative effect of LCL161 was confirmed in some solid tumors. For example, LCL161 sensitized radiotherapy through inhibiting the expression of cIAP1, activating caspase-8 and leading to apoptosis in esophageal cancer cells [17]. In liver cancer cells, LCL161 improved the sensitivity of paclitaxel to drug-induced cell death, and inhibition of Bcl-2 reversed LCL161 resistance [18, 19]. However, there are no reports of the use of LCL161 in lung cancer. Therefore, we investigated use of LCL161 alone and in combination with paclitaxel in lung cancer both in vitro and in vivo.

In this study, we found that the combination of LCL161 and paclitaxel inhibited tumor growth both in vitro and in vivo. Paclitaxel induced cell apoptosis by activating caspase-8 and caspase-3. When LCL161 was combined with paclitaxel, it degraded cIAP1 and cIAP2, which are highly expressed in NSCLC patients and confer a poor prognosis, and it resulted in the release of RIP1 from the TRADD-TRAF2-RIP1-cIAP complex, leading to the formation of the RIP1-FADD-Caspase-8 complex. Subsequently, caspase-8 and caspase-3 were activated, ultimately leading to cell apoptosis.

## Methods

### Patients and samples

All specimens were collected from 126 NSCLC patients (IA-IIIA; 32 females and 94 males; age range: 33–79 years) who underwent pulmonary surgery between October 2006 and December 2009 at the First Affiliated Hospital of Xi’an Jiaotong University (Xi’an, China). Clinicopathological data, including gender, age, histology, tumor differentiation, tumor location, tumor size, lymph node metastasis, pTNM stage and overall survival, were collected for all cases. All patients had a single tumor without distant metastasis, and none of them had previously been treated with chemo- or radiotherapy. The study was approved by the Ethics Committee of the First Affiliated Hospital of Xi’an Jiaotong University, based on the patients’ written informed consent for the usage of the biologic material.

### Immunohistochemistry staining

Tumor specimens were fixed with 10 % formaldehyde and embedded in paraffin blocks. Sections (4 μm) were deparaffinized with xylene, rinsed and rehydrated through a graded series of alcohols. Hematoxylin and eosin staining was used to confirm the original histopathological diagnosis. For immunohistochemistry, the slides were treated with 3 % H_2_O_2_ for 10 min and then immersed in 0.01 M citrate buffer (pH 6.0) for 3 min in a pressure cooker at 125 °C. After washing with PBS, the slides were incubated with diluted primary antibody (anti-cIAP1, cIAP2, and active caspase-3 from Abcam, Cambridge, UK, and anti-Ki67 from Cell Signaling Technology, MA, USA) at 4 °C overnight and IgG/HRP secondary antibody (1:250; Beijing Biosynthesis Inc., Beijing, China) for 30 min at room temperature. The reaction was developed using a DAB chromogen solution. Counterstaining was performed with hematoxylin before dehydration and mounting. Staining reactions were determined by microscopic examination. Both the intensity and extent of staining were considered when analyzing the data. The extent of staining was scored from 0 to 100 % (1 indicates 1–25 %, 2 indicates 26–50 %, 3 indicates 51–75 %, 4 indicates 76–100 %), and the intensity of staining was scored from 0 to 2 (0 indicates none; 1 indicates weak to moderate; 2 indicates strong). The IHC score was determined as follows: high expression (+): score ≥ 3; low expression (−): score ≤ 2.

### Cell culture, drug treatment and transfection

The human cell lines A549 and H460 were purchased from American Type Culture Collection (ATCC, VA, USA). All cell lines were cultured in DMEM + 10 % FBS (GE Healthcare Life Sciences, UT, USA) at 37 °C in a humidified atmosphere consisting of 5 % CO_2_ and 95 % air. For drug treatment, cells were seeded into 6- to 96-well plates and treated with a range of concentrations of LCL161 and/or paclitaxel (Sigma, MO, USA) before detection. The siRNAs for cIAP1,2 or RIP1 and the control siRNAs were designed and produced by the Shanghai GenePharma Co., Ltd (Shanghai, China). The siRNAs or control siRNAs were transfected into NSCLC cells with Lipofectamine 2000 (Invitrogen Life Technologies, CA, USA). Western blotting was used to identify protein knockdown in cells.

### Cell viability assay (MTT)

Cell viability was determined by the MTT assay. Cells (10^4^ cells/well) were seeded in 96-well plates with 200 μl of DMEM containing 10 % FBS. After 24 h, the cells were exposed to LCL161 and/or paclitaxel at different concentrations for 48 h. Next, 20 μl of MTT was added per well at 37 °C for 4 h. The purple formazan crystals were dissolved in 200 μl of DMSO. After 15 min, the absorbance was measured at 490 nm with a microplate reader (Bio-Rad Laboratories, CA, USA).

### Apoptosis assay

Apoptosis was determined using an apoptosis detection kit (BD Biosciences, NJ, USA). After washing with PBS, 10^6^ cells were resuspended in 100 μl binding buffer, followed by incubation with 5 μl FITC-Annexin V and 5 μl PI for 20 min at room temperature in the dark. Next, 400 μl of binding buffer was added, and flow cytometry was performed using a FACScan flow cytometer (BD Biosciences).

### Caspase-8 and caspase-3 activity assays

Cells were washed with PBS and resuspended in lysis buffer on ice for 15 min. After centrifugation, the supernatants were collected and the caspase-8 and caspase-3 activities were measured using the Caspase-8 Activity Assay Kit and the Caspase-3 Activity Assay Kit (Beyotime, Shanghai, China) according to the manufacturer’s instructions. The working principle of this kit is based on the cleavage of the caspase-8 substrate, Ac-IETD-pNA, and the caspase-3 substrate, Ac-DEVD-pNA. The release of p-nitroanilide (pNA) was qualified by determining the absorbance at 405 nm using a microplate reader. Caspase-8 and caspase-3 activities were expressed relative to control cells.

### Western blot and Co-immunoprecipitation assay

Western blot and co-immunoprecipitation assay were performed as described previously [20] using the following antibodies: Survivin, anti-FADD, pro-caspase-8 (Santa Cruz Biotechnology, CA, USA), XIAP (Epitomics, CA, USA), cleaved Caspase-8, TNFα, TRAF2, TRADD (Cell Signaling Technology). cIAP1, cIAP2, RIP1, TNFR1 and cleaved Caspase-3 and β-Actin (Abcam, Cambridge, UK) was used as a loading control. Proteins were detected using ECL detection (Thermo scientific, MA, USA). All western blot data shown are representative of at least three independent experiments.

### Enzyme Linked Immunosorbent Assay (ELISA)

The media for H460 and A549 cell cultures were collected 24 h after the paclitaxel treatment. The level of TNFα in the cell culture media was quantified using the Human TNFα ELISA Kit (Beijing Biosynthesis Inc., Beijing, China) in accordance with the manufacturer’s instructions and measured at 450 nm in a microplate reader.

### Nude mouse xenograft studies

Four-week-old BALB/c (athymic) nude mice were purchased from the Center of Laboratory Animals of Xi’an Jiaotong University and were bred under specific-pathogen-free conditions. All animal experiments were approved by the Ethics Committee of the First Affiliated Hospital of Xi’an Jiaotong University. A total of 5 × 10^6^ H460 cells were subcutaneously injected into the right flank of nude mice. When the average tumor volume reached 100 mm^3^, the mice were randomly divided into 4 groups. We treated the mice with 10 mg/kg LCL161 and/or 20 mg/kg paclitaxel through intraperitoneal injection every two days for three weeks. The tumor size and body weight were recorded with a caliper using the following formula: tumor volume = length × width × width/2. One week later, the mice were sacrificed, and solid tumors were removed for further analyses.

### Statistical analysis

The results are presented as the mean values ± SD. Pearson chi-squared and Fisher’s exact tests were used to compare frequencies. The correlation between the expression of cIAP1 and cIAP2 was evaluated by Spearman correlation analysis. Kaplan–Meier analysis was used to produce survival curves with differences tested between groups by the log-rank test. Univariate and multivariate analyses were performed with the Cox regression model. Statistical significance was assessed by two-tailed Student’s *t* test. Statistical analyses were performed using SPSS version 13.0 (SPSS, Chicago, IL, USA). *P* < 0.05 was considered to indicate a statistically significant result.

## Results

### cIAP1 and cIAP2 expression in NSCLC and para-tumor tissues

**Fig. 1:**
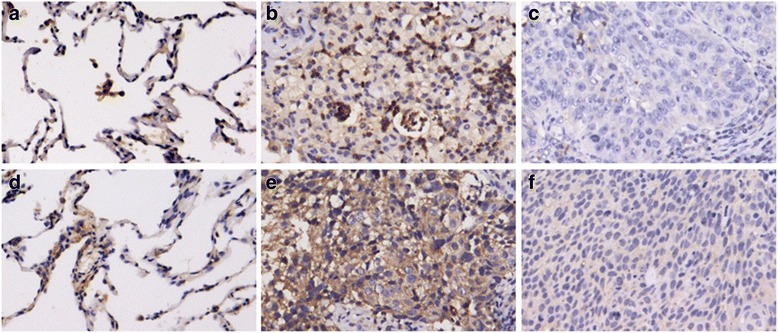
The expression of cIAP1 and cIAP2 in NSCLC and para-tumor tissues by immunohistochemistry. The positive expression of cIAP1 (**a**) and cIAP2 (**d**) in para-tumor tissues. The positive (**b**) and negative (**c**) expression of cIAP1 in NSCLC tissues. The positive (**e**) and negative (**f**) expression of cIAP2 in NSCLC tissues. Original magnification, ×200

Immunohistochemistry was performed to characterize the expression of cIAP1 and cIAP2 in 126 NSCLC tissues and 102 para-tumor tissues. The results showed that, in the NSCLC tissues, cIAP1 was usually identified in both the cytoplasm and nucleus, while cIAP2 was identified only in the cytoplasm (Fig. 1). Of the 126 patients, tumors from 82 (65.1 %) were positive for cIAP1, and tumors from 58 (46.0 %) were positive for cIAP2. Of the 102 para-tumor tissues, 16 (15.7 %) were positive for cIAP1, and 19 (18.6 %) were positive for cIAP2. The expression of cIAP1 and cIAP2 in NSCLC was significantly higher than that in the para-tumor tissues (*P* < 0.001) (Table 1). Tumors from 31 (24.6 %) patients expressed high levels of cIAP1 and low levels of cIAP2. Tumors from 7 (5.6 %) patients expressed low levels of cIAP1 and high levels of cIAP2. Tumors from 51 (40.5 %) patients expressed high levels of both cIAP1 and cIAP2, whereas tumors from 37 (29.4 %) patients expressed low levels of both proteins. Moreover, a positive correlation was found between cIAP1 and cIAP2 expression in NSCLC (*r*_s_ = 0.443, *P* < 0.001) (Table 2).

**Table 1:** Comparison of cIAP1, cIAP2 expression in NSCLC and para-tumor tissues

Variable	*n*	c-IAP1		*P*	c-IAP2		*P*
	(%)	Negative	Positive		Negative	Positive	
NSCLC	126	44(34.9)	82(65.1)	<0.001	68(54.0)	58(46.0)	<0.001
para-tumor	102	86(84.3)	16(15.7)		83(81.4)	19(18.6)

**Table 2:** Correlation of cIAP1 and cIAP2 expression in NSCLC

	c-IAP2		*r*	*P* value
	Negative	Positive		
c-IAP1				
Negative	37(29.4)	7(5.6)	0.443	<0.001
Positive	31(24.6)	51(40.5)		

### cIAP1/cIAP2 expression and clinicopathological characteristics

The contingency table analysis was used to examine the correlation between cIAP1/cIAP2 expression and clinicopathological characteristics in NSCLC patients. The associations between clinicopathological variables and cIAP1/cIAP2 expression are shown in Table 3. Positive cIAP1 expression was found to be significantly associated with pTNM stage (*P* = 0.025) but not with gender, age, histology, tumor differentiation, tumor location, tumor size, or lymph node metastasis (*P* > 0.05). However, there was no significant association between cIAP2 and any parameter (*P* > 0.05).

**Table 3:** Correlation between cIAP1, cIAP2 expression and clinicopathological characteristics

Variable	*n*	c-IAP1		*P*	c-IAP2		*P*
		Negative	Positive		Negative	Positive	
Gender							
Male	94	32	62	0.723	50	44	0.764
Female	32	12	20		18	14	
Age							
≤ 65	79	28	51	0.873	44	35	0.614
> 65	47	16	31		24	23	
Histology							
Squamous cell carcinoma	51	19	32	0.661	30	21	0.665
Adenocarcinoma	59	21	38		30	29	
Others	16	4	12		8	8	
Tumor differentiation							
G1	15	7	8	0.591	8	7	0.176
G2	67	22	45		41	26	
G3	44	15	29		19	25	
Tumor location							
central type	69	24	45	0.971	40	29	0.321
peripheral type	57	20	37		28	29	
Tumor size							
T1	32	13	19	0.423^a^	22	10	0.074^a^
T2	77	28	49		39	38	
T3	14	3	11		7	7	
T4	3	0	3		0	3	
Lymph node metastasis							
N0	74	30	44	0.126	44	30	0.115
N1	29	10	19		16	13	
N2	23	4	19		8	15	
pTNM stage							
I	57	26	31	0.025*	34	23	0.084
II	43	14	29		25	18	
III	26	4	22		9	17	
Total	126	44	82		68	58	

^a^Fisher’s exact tests; *p < 0.05

### Prognostic value of cIAP1 and cIAP2 expression

**Fig. 2:**
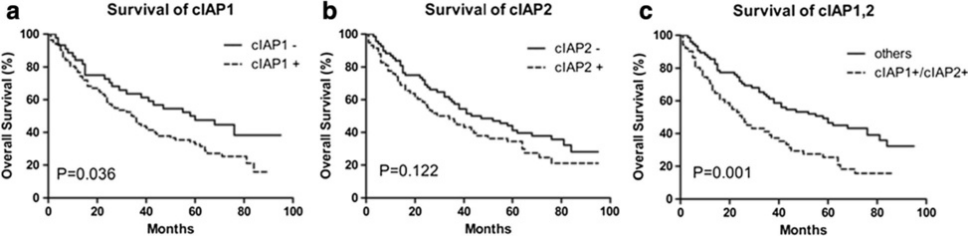
Association between the expression of cIAP1 or cIAP2 and the survival of patients with NSCLC. Overall survival was plotted as a function of **a** cIAP1 expression (log rank, *P* = 0.036), **b** cIAP2 expression (log rank, *P* = 0.122), and **c** cIAP1 and cIAP2 expression (log rank, *P* = 0.001). Analysis was performed by the Kaplan–Meier method. The log-rank test was used to compare survival curves

Associations between cIAP1/cIAP2 expression and overall survival were evaluated by Kaplan–Meier survival curves and the log-rank test. The patients whose tumors were positive for cIAP1 had a significantly longer overall survival than those whose tumors were negative for cIAP1(Log-rank = 4.378, *P* = 0.036) (Fig. 2a). However, the prognosis of patients whose tumors were positive for cIAP2 did not differ from those who were cIAP2 negative (Log-rank = 2.396, *P* = 0.122) (Fig. 2b). To better evaluate the prognostic value of cIAP1 and cIAP2 expression, the OS of patients whose tumors were positive for both cIAP1 and cIAP2 (cIAP1+/cIAP2+) was evaluated. This group demonstrated a significantly shorter OS than the rest of the patients (cIAP1-/cIAP2+, cIAP1+/cIAP2-, and cIAP1-/cIAP2- combined; Log-rank = 10.29, *P* = 0.001), and this difference was much more obvious compared to the OS differences between patients who were positive or negative for cIAP1 or cIAP2 alone (cIAP1+ vs. cIAP1-, *P* = 0.036; cIAP2+ vs. cIAP2-, *P* = 0.122) (Fig. 2c).

In univariate analysis based on the Cox regression model, we analyzed the predictive value of cIAP1 and cIAP2 expression, as well as that of other clinicopathological characteristics, including gender, age, and histology. The results indicated that tumor size, lymph node metastasis, pTNM stage, cIAP1 expression, and cIAP1+/ cIAP2+ were good predictors of OS for NSCLC patients (Table 4). The pTNM stage of NSCLC is dependent on tumor size and lymph node metastasis; therefore, we analyzed other predictive factors, including tumor size, lymph node metastasis and cIAP1. cIAP1 was not an independent predictive factor for the prognosis of NSCLC. In multivariate Cox regression analysis by tumor size, lymph node metastasis and cIAP1+/ cIAP2+, the results revealed that cIAP1+/ cIAP2+ has a relative risk of 1.625 for OS, with *P* = 0.029 (Table 5).

**Table 4:** Univariate Cox regression analysis of overall survival in NSCLC patients

Variable	HR	95 % CI	*P*-value
Gender	0.93	0.578–1.497	0.765
Age	0.968	0.628–1.493	0.884
Histology	1.015	0.747–1.379	0.926
Tumor differentiation	1.275	0.923–1.763	0.141
Tumor location	1.008	0.662–1.536	0.97
Tumor size	1.684	1.180–2.404	0.004*
Lymph node metastasis	1.891	1.460–2.451	<0.001*
pTNM stage	2.141	1.630–2.811	<0.001*
cIAP1	1.623	1.024–2.572	0.039*
cIAP2	1.388	0.912–2.111	0.126
cIAP1+/ cIAP2+	1.965	1.287–3.000	0.002*

*HR* hazard ratio, *CI* confidence interval, *p < 0.05

**Table 5:** Multivariate Cox regression analysis of overall survival in NSCLC patients

Variable	B	SE	Wald	*P*-value	HR	95 % CI
Tumor size	0.359	0.183	3.858	0.050	1.432	1.001–2.049
Lymph node metastasis	0.545	0.135	16.160	0.000*	1.724	1.322–2.248
cIAP1	0.331	0.238	1.933	0.164	1.393	0.873–2.222
Tumor size	0.328	0.183	3.209	0.073	1.388	0.970–1.986
Lymph node metastasis	0.530	0.135	15.300	0.000*	1.698	1.302–2.215
cIAP1+/ cIAP2+	0.485	0.222	4.792	0.029*	1.625	1.052–2.510

*B* partial regression coefficient, *S.E*. standard error of partial regression coefficient, *Wald X* ^2^ value which was used to compare if there was difference between total partial regression coefficient and 0, *HR* hazard ratio, *CI* confidence interval, *p < 0.05

Taken together, cIAP1 expression is an independent factor that can be used to evaluate prognosis in NSCLC patients, with cIAP1 expression predicting a poorer prognosis, especially in patients whose tumors are positive for cIAP2.

### LCL161 and paclitaxel synergistically reduce cell viability and induce cell apoptosis in NSCLC cells

**Fig. 3:**
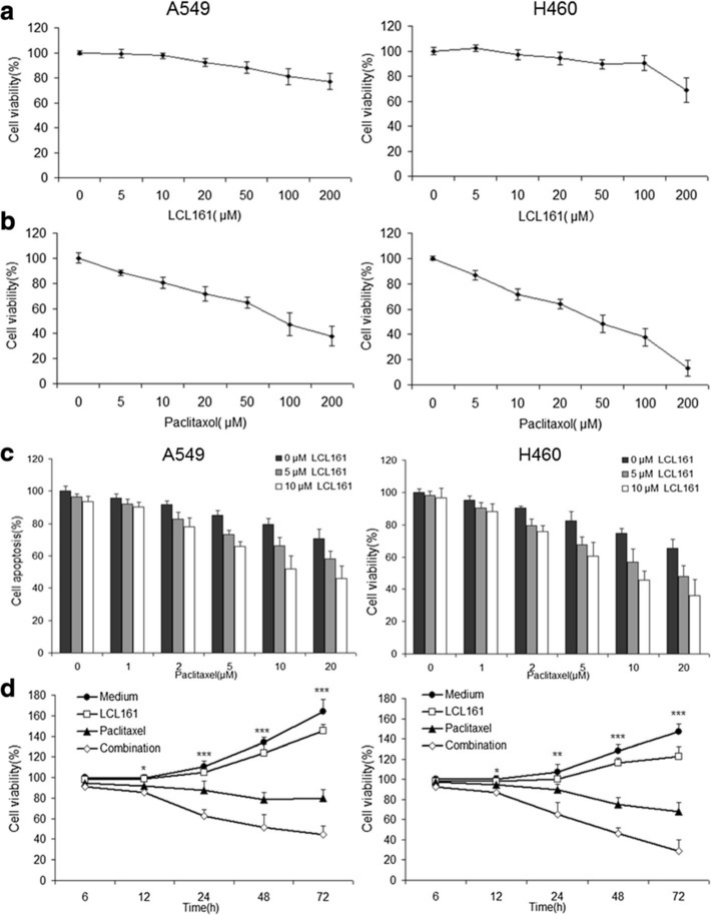
LCL161 and paclitaxel synergize to reduce cell viability in NSCLC cells. A549 and H460 lung cancer cells were treated for 48 h with the indicated concentrations of LCL161 (**a**) or paclitaxel (**b**). Cells were treated for 48 h with the indicated concentrations of LCL161 and paclitaxel (**c**) or for the indicated times with 10 μM LCL161 and/or 10 μM paclitaxel (**d**). Cell viability was determined by the MTT assay. Data are represented as mean ± SD; *P < 0.05; **P < 0.01; ***P < 0.001

**Fig. 4:**
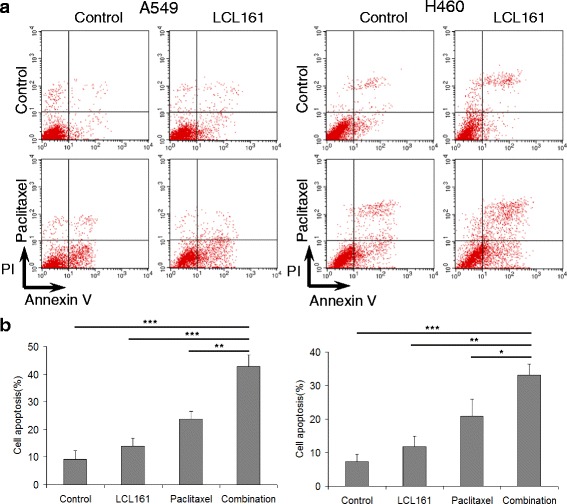
LCL161 and paclitaxel synergistically induce cell apoptosis in NSCLC cells. **a** A549 and H460 lung cancer cells were treated with 10 μM LCL161 and/or 10 μM paclitaxel for 48 h. Annexin V/PI staining was used to detect apoptosis. **b** Statistical analysis of the proportion of lung cancer cells in different periods. Data are represented as mean ± SD; *P < 0.05; **P < 0.01; ***P < 0.001

The antiproliferative effects of LCL161 and paclitaxel were evaluated by MTT assays. A549 and H460 cells were treated with 0–200 μM LCL161 or paclitaxel for 48 h. Cells viability was reduced prominently with paclitaxel treatment but not with LCL161 treatment (Fig. 3a, b). When cells were treated with a combination of 0–10 μM LCL161 and 0–20 μM paclitaxel, the cell viability was lower than with paclitaxel treatment alone (Fig. 3c). Additionally, cells treated with 10 μM LCL161 and/or 10 μM paclitaxel for 6–72 h showed time-dependent viability (Fig. 3d). To further study the apoptotic effects of the combination, we treated cells with 10 μM LCL161 and/or 10 μM paclitaxel for 48 h, and cell apoptosis was measured by Annexin V/PI analysis. Consistent with the results of the MTT assay, cell apoptosis in the LCL161/paclitaxel co-treatment group was significantly decreased compared with that in cells treated with LCL161 or paclitaxel alone (*P* < 0.05, Fig. 4a, b).

### Paclitaxel increases TNFα secretion, and LCL161 decreases the expression of cIAP1 and cIAP2

**Fig. 5:**
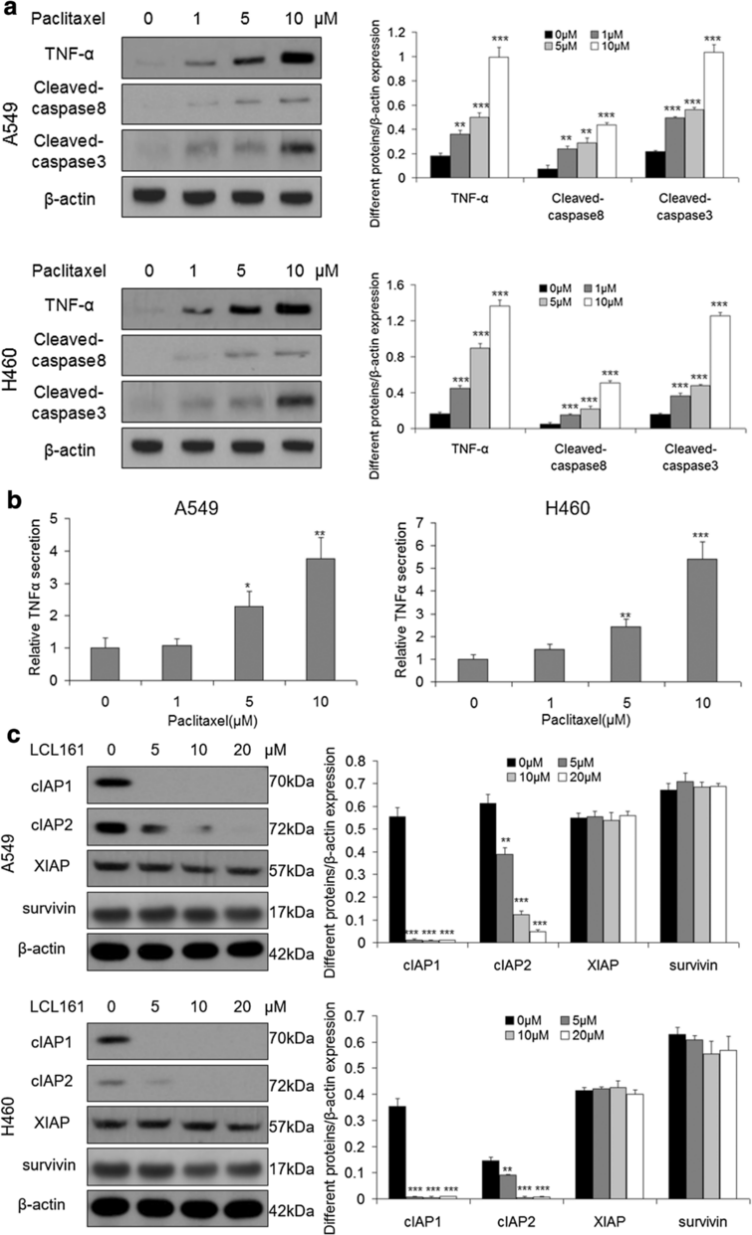
Paclitaxel increases TNFα expression, and LCL161 decreases cIAP1 and cIAP2 expression. **a** A549 and H460 cells were treated for 48 h with 0–10 μM paclitaxel. The protein levels of TNF-α and cleaved caspase-8 and cleaved caspase-3 were assessed by western blotting. **b** Cells were treated for 24 h with 0–10 μM paclitaxel. The level of TNFα in the cell culture media was quantified using the Human TNFα ELISA Kit. **c** A549 and H460 cells were treated for 48 h with 0–20 μM LCL161. The protein levels of cIAP1, cIAP2, XIAP and survivin were assessed by western blotting. β-actin was used as a loading control. The bar graphs represent the mean ± SD of different proteins/β-actin; *P < 0.05; **P < 0.01; ***P < 0.001

**Fig. 6:**
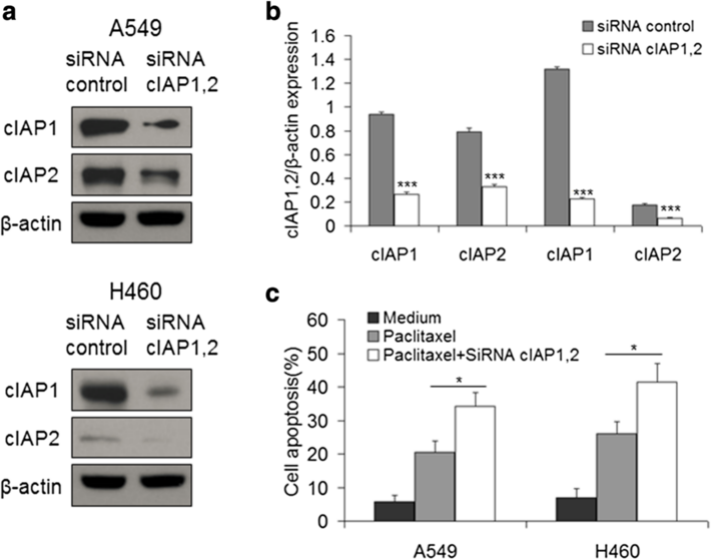
The knockdown of cIAP1 and cIAP2 increases paclitaxel-induced cell apoptosis. **a** A549 and H460 cells were transfected with siRNA control or siRNA cIAP1,2 for 48 h. The expression of cIAP1,2 was assessed by western blotting. β-Actin was used as a loading control. **b** The bar graphs represent the mean ± SD of cIAP1 or cIAP2/β-actin. **c** After transfection with siRNA for 48 h, the cells were treated for 48 h with 10 μM paclitaxel, and their apoptosis was detected by Annexin V/PI staining. Data are represented as mean ± SD; *P < 0.05; **P < 0.01; ***P < 0.001

It has been reported that Smac mimetics induce TNFα-dependent cancer cell death by targeting IAPs. To investigate whether paclitaxel promotes LCL161-induced apoptosis via TNFα, western blotting was performed after cells were treated with 0–10 μM paclitaxel alone for 48 h. The expression of TNFα increased coincident with the activation of caspase-8 and caspase-3 during paclitaxel treatment (Fig. 5a). In addition, for quantification of secreted TNFα, supernatants were collected after treatment with paclitaxel and analyzed by ELISA. Paclitaxel treatment increased TNFα secretion in lung cancer cells (Fig. 5b). Besides, cells were treated with 0–10 μM LCL161 alone for 48 h, and IAPs were detected by western blotting. The expression of XIAP and Survivin did not change, whereas that of cIAP1 and cIAP2 was significantly decreased (*P* < 0.01, Fig. 5c). Additionally, we used siRNA to knockdown cIAP1 and cIAP2 and then treated the cells with paclitaxel. The knockdown of cIAP1 and cIAP2 was verified by western blotting (Fig. 6a, b). As expected, the paclitaxel-induced apoptosis was significantly increased after knockdown of cIAP1 and cIAP2 (*P* < 0.05, Fig. 6c).

### LCL161 and paclitaxel cooperate to activate caspase-3 and caspase-8

**Fig. 7:**
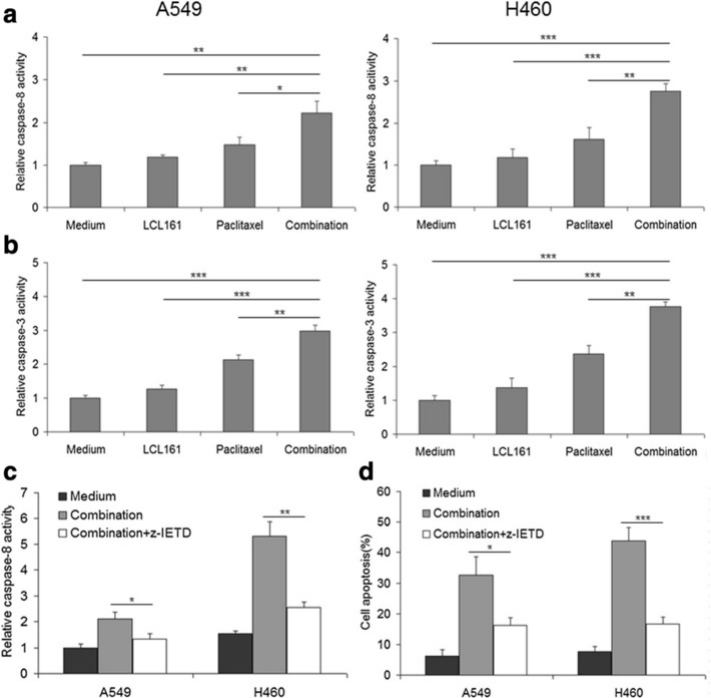
LCL161 and paclitaxel cooperate to activate caspase-3 and caspase-8. A549 and H460 cells were treated for 24 h with 10 μM LCL161 and/or 10 μM paclitaxel. The changes in caspase-8 (**a**) and caspase-3 (**b**) activity were determined as described in the Materials and Methods section. Data are provided as mean ± SD, *P < 0.05; **P < 0.01; ***P < 0.001. **c** NSCLC cells were treated for 24 h with the combination of 10 μM LCL161 and 10 μM paclitaxel in the presence or absence of 20 μM caspase-8 inhibitor Z-IETD-FMK. Caspase-8 activity was detected using a caspase-8 activity assay kit. **d** Annexin V/PI staining was used to detect apoptosis, and the apoptotic rates were provided as mean ± SD; *P < 0.05; **P < 0.01; ***P < 0.001

We used caspase activity assays to further confirm the function of caspase-3 and caspase-8 in apoptosis induced by LCL161/paclitaxel co-treatment. The co-treatment significantly increased caspase-3 and caspase-8 activity in both A549 and H460 cells (*P* < 0.01, Fig. 7a, b). To confirm whether caspase-8 activation is indispensable in cell apoptosis induced by LCL161 and paclitaxel, we used the caspase-8 inhibitor Z-IETD-FMK. The addition of Z-IETD-FMK significantly inhibited caspase-8 activation and decreased cell apoptosis during LCL161/paclitaxel co-treatment (*P* < 0.05, Fig. 7c, d). These experiments demonstrated that LCL161 and paclitaxel cooperate to trigger caspase-8 and caspase-3 activation, thereby inducing apoptosis.

### RIP1 is a critical mediator of LCL161/paclitaxel-induced apoptosis

**Fig. 8:**
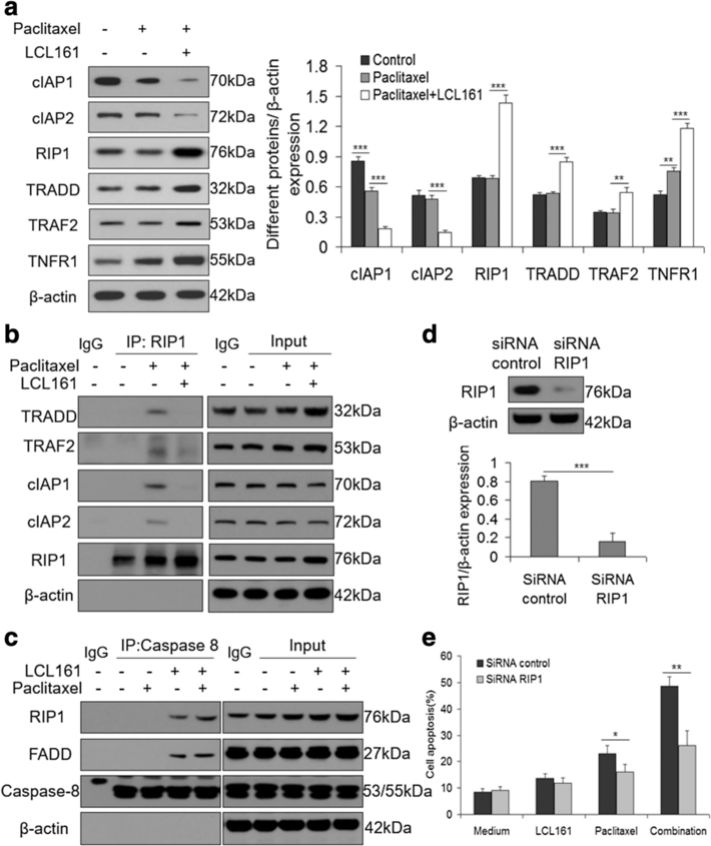
RIP1 is a critical mediator of LCL161-induced apoptosis. **a** A549 cells were treated for 48 h with 10 μM paclitaxel with/without 10 μM LCL161, and western blotting was performed for TRADD, TRAF2, RIP1, TNFR1, cIAP1 and cIAP2. **b** A549 cells were treated with 10 μM paclitaxel with/without 10 μM LCL161 for 48 h, and 5 μM proteasome inhibitor MG132 for 4 h before collecting the cells. Anti-RIP1 antibody was used to co-immunoprecipitate RIP1 and detected its interaction with TRADD, TRAF2 and cIAP. **c** A549 cells were treated for 48 h with 10 μM LCL161 and/or 10 μM paclitaxel in the presence of 20 mM Z-IETD-FMK. Caspase-8 was immunoprecipitated using an anti-caspase-8 antibody. The detection of RIP1 and FADD proteins was performed by western blot analysis. **d** A549 cells were transiently transfected with siRNA sequences against RIP1 for 48 h and then were treated with 10 μM LCL161 and/or 10 μM paclitaxel for another 48 h. RIP1 expression was analyzed by western blotting 48 h post-transfection. **e** Annexin V/PI staining was used to detect apoptosis, and the apoptotic rates are provided as mean ± SD; *P < 0.05; **P < 0.01; ***P < 0.001

As Smac mimetics have been shown to release RIP1 from the TRADD-TRAF2-RIP1-cIAP complex, we assessed the changes in the levels of these proteins during treatment with paclitaxel and/or LCL161. Paclitaxel decreased the expression of cIAP1 but not TRADD, TRAF2, RIP1 and cIAP2. However, LCL161 accelerated the degradation of cIAP1 and cIAP2, whereas it led to the accumulation of TRADD, TRAF2, and RIP1. Besides, TNFR1 was increased during treatment with paclitaxel and/or LCL161 (Fig. 8a). Furthermore, using co-immunoprecipitation, we found that RIP1 directly interacted with TRADD, TRAF2 and cIAP in the presence of paclitaxel treatment and that RIP1 was released from the complex during LCL161/paclitaxel co-treatment (Fig. 8b).

Next, we assessed whether the formation of the RIP1-FADD-Caspase-8 complex, after RIP1 release from the TRADD-TRAF2-RIP1-cIAP complex, caused caspase-8 activation. We used anti-caspase-8 antibody to co-immunoprecipitate caspase-8 and detected its interaction with FADD and RIP1 in A549 cells. As expected, caspase-8 directly interacted with RIP1 and FADD in the presence of LCL161 with or without paclitaxel (Fig. 8c).

To investigate the requirement for RIP1 in LCL161/paclitaxel-induced apoptosis in A549 cells, we transiently silenced RIP1 with a small interfering RNA and treated cells with 10 μM LCL161 and/or 10 μM paclitaxel for 24 h, followed by staining with propidium iodide and FITC-Annexin V to assess apoptosis by flow cytometry. In the RIP1 knockdown A549 cells, LCL161/paclitaxel-induced apoptosis was significantly attenuated compared with that in control cells treated with the same drugs (*P* < 0.01, Fig. 8d, e). Together, this set of experiments demonstrates that RIP1 is a critical mediator of LCL161/paclitaxel-induced caspase-8 activation and apoptosis.

### The combination of LCL161 and paclitaxel inhibits tumor growth by degrading cIAP1 and cIAP2 and activating caspase-3 in vivo

**Fig. 9:**
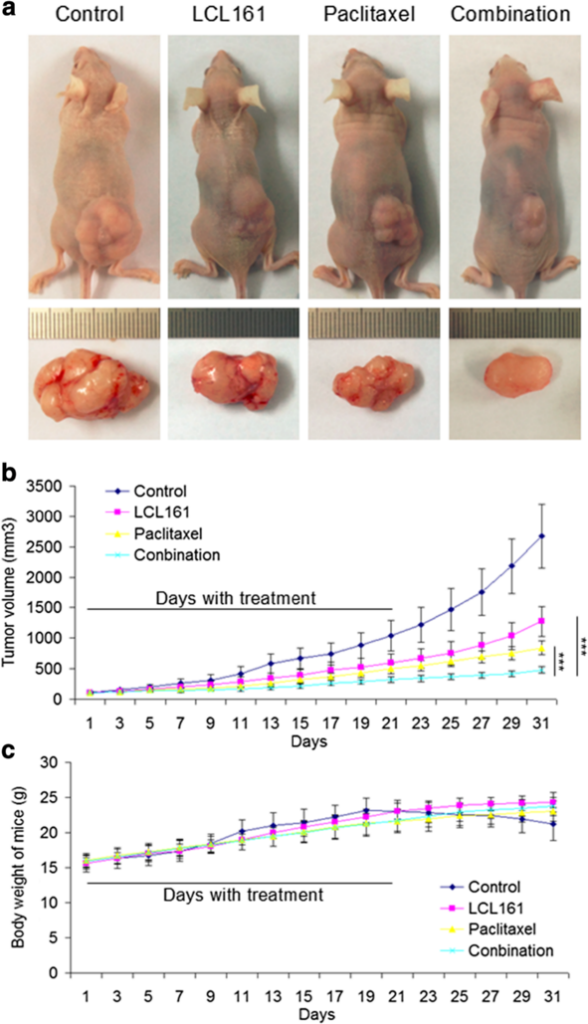
The combination of LCL161 and paclitaxel has strong antitumor activity in vivo. Approximately 2 × 10^6^ H460 cells were injected subcutaneously into each nude mouse. After tumor formation, 10 mg/kg LCL161 and/or 20 mg/kg paclitaxel were intraperitoneally injected into the mice. **a** Typical images of mice with H460 xenografts in the control, LCL161, paclitaxel and drug combination groups. Curves of tumor growth (**b**) and body weight (**c**) for H460 xenografts with drug treatment. The tumor volume and body weight of the mice are provided as mean ± SD; *P < 0.05; **P < 0.01; ***P < 0.001

We next sought to determine whether LCL161 and paclitaxel affect tumor growth, by using an H460 subcutaneous tumor model. Representative images of xenografts from mice treated with LCL161 and/or paclitaxel for three weeks are shown in Fig. 9a. Compared with the control group, the tumor volume was smaller following treatment with LCL161 or paclitaxel (*P* < 0.001, Fig. 9b). Additionally, the drug combination manifested much better antitumor activity than either LCL161 or paclitaxel alone (*P* < 0.001, Fig. 9b). Furthermore, there was no prominent weight loss in mice treated with LCL161, paclitaxel, or the combination (*P* > 0.05, Fig. 9c).

**Fig. 10:**
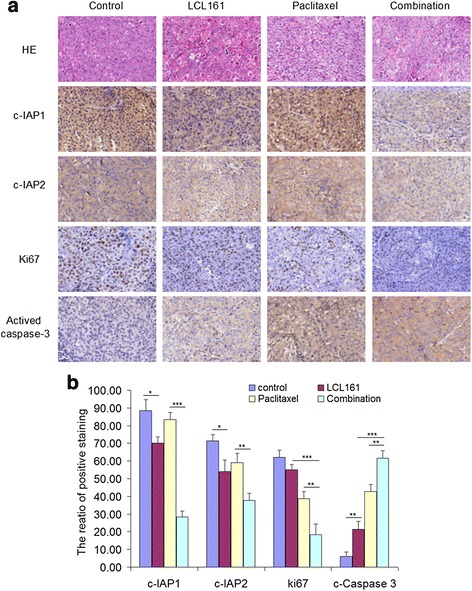
The combination of LCL161 and paclitaxel inhibits cIAP1 and cIAP2 and activates caspase-3 in vivo. **a** The histology of tumor xenograft tissues was confirmed using hematoxylin and eosin (H&E) staining. cIAP1, cIAP2, Ki67 and activated caspase-3 were detected by immunohistochemistry. Original magnification, ×200. **b** Quantification of the ratio of the positive staining in each tumor tissue region, provided as mean ± SD; *P < 0.05; **P < 0.01; ***P < 0.001

We performed H&E staining to verify tumor tissues and immunohistochemistry to detect the expression of cIAP1, cIAP2, caspase-3 and Ki-67 in the xenografted tissues (Fig. 10a). As expected, LCL161 significantly down-regulated cIAP1 and cIAP2 and activated caspase-3 in vivo (*p* < 0.05), especially in the groups that received the drug combination (*p* < 0.01, Fig. 9b). Decreased expression of Ki-67 was observed in the LCL161 and paclitaxel co-treatment group, indicating that tumor cell proliferation was significantly inhibited by drugs co-treatment (*p* < 0.01, Fig. 10b). Taken together, these data indicate that the combination of LCL161 and paclitaxel inhibits tumor growth by degrading cIAP1 and cIAP2 and activating caspase-3 in a NSCLC xenograft model.

## Discussion

IAPs are highly expressed in numerous tumor tissues and their levels are closely related to the prognosis of patients [7, 8]. The overexpression of cIAP1 in bladder cancer, cervical cancer, and head and neck tumors indicates a poor prognosis, while the overexpression of cIAP2 is a biomarker for the early advanced stage of pancreatic cancer [21–23]. In lung cancer studies, expression of cIAPl and cIAP2 was correlated but they didn’t predict response to chemotherapy [24]. cIAPl mRNA expression was elevated in patients with adenocarcinoma, especially in low TMN adenocarcinomas [25]. cIAP2 were increased in more advanced grades of bronchial IEN lesions than in normal bronchial epithelium [26]. In this study, we demonstrated that the expression of cIAP1 and cIAP2 in resectable NSCLC was higher than that in adjacent normal tissues. cIAP1 was highly expressed in patients with late TNM stage and a poor prognosis, but the *P*-value (0.036) was only slightly lower than 0.05. When analyzing the prognosis by combining cIAP1 with cIAP2, we found that the prognosis was even worse in patients with high expression of both cIAP1 and cIAP2, and the *P*-value was less than 0.001 compared with the rest of patients. Therefore, it was more effective to evaluate prognosis by combining cIAP1 with cIAP2, possibly because cIAP1 and cIAP2 have similar structures and therefore a similar capacity to inhibit apoptosis, and it was more difficult to induce cell apoptosis if both of these were highly expressed. Actually, more than forty percent of tumor expressed a high level of both cIAP1 and cIAP2. It probably because a potential proto-oncogene, 11q21-23, encodes both cIAP1 and cIAP2, and expresses high in tumor tissues [7]. Besides, some studies revealed that nuclear expression of cIAP1 was strongly correlated to poor patient prognosis in bladder cancer and head and neck squamous cell carcinomas [21, 27]. The function of nuclear cIAP and the mechanism of its degradation under treatment with Smac mimetics are still unknown. So, it could be interesting to quantify and analyze the nuclear cIAP in NSCLC patients and try to explore its mechanisms under treatment with Smac mimetics in the future.

Many studies have shown that Smac mimetics can activate caspases and the apoptotic pathway by degrading IAPs, especially cIAP1 and cIAP2, leading to apoptosis [28, 29]. In our study, we demonstrated that LCL161 sensitized paclitaxel by degrading IAPs. In addition, paclitaxel increased the expression of TNFα and activated the exogenous apoptotic pathway, while LCL161 degraded IAPs, activated caspase-8 in the pathway and induced cell apoptosis. The latter finding explains why the effect of LCL161 is not obvious when used alone but is very prominent in combination with paclitaxel. This was also consistent with other studies showing that Smac mimetics can induce apoptosis when combined with TNFα [30, 31]. Furthermore, we knocked down cIAP1 and cIAP2 expression with siRNA, sensitizing cells to paclitaxel-induced apoptosis. Additionally, the caspase-8 inhibitor Z-IETD-FMK was able to inhibit apoptosis in cells co-treated with paclitaxel and LCL161. These results further show that the two-drug combination induced cell apoptosis by degrading cIAP1 and cIAP2 and activating caspase-8, thereby inducing the exogenous apoptotic pathway. It has been shown that the caspase 8 inhibitor, FLIP, inhibited Smac induced apoptosis and down-regulation of FLIP enhanced Smac mimetic induced cell death [12, 32, 33]. Besides, some other studies showed that FLIP was a target for paclitaxel/taxol induced apoptosis [34–36]. This is probably another mechanism of LCL161 and paclitaxel combination induced cell apoptosis.

**Fig. 11:**
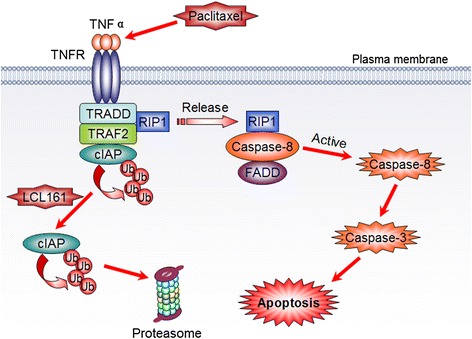
LCL161 increases paclitaxel-induced apoptosis by targeting cIAPs. Paclitaxel activates the TNFα pathway, leading to the recruitment of various factors and the formation of the TRADD-TRAF2-RIP1-cIAP complex. LCL161 degrades cIAPs by increasing cIAP self-ubiquitination, which results in the release of RIP1, the formation of the caspase-8/RIP1/FADD complex, and the activation of caspase-8 and, ultimately, apoptosis

To further explore the mechanisms of LCL161- and paclitaxel-induced apoptosis, we investigated RIP1 complex to connect cIAP1/2 with caspase-8. We found that paclitaxel promoted RIP1 binding to TRADD, TRAF2 and cIAP1/2. When LCL161 was combined with paclitaxel, cIAP1 and cIAP2 were degraded, but other proteins accumulated. RIP1 (and also TRAF2) is ubiquitination targets of cIAP1. cIAP1 can induce the conjugation of K63-ubiquitin chains promoting the activation of NF-kB and also K48-ubiquitin chain inducing proteasomal degradation of RIP1 (and TRAF2) [37, 38]. Thus, the upregulation of RIP1 (and TRAF2) could be related to an inhibition of UPS-mediated degradation, because of cIAPs degradation. On co-treatment with paclitaxel and LCL161, TNFα pathway is activated and cIAPs are ubiquitinated, therefore, RIP1 and TRAF2 were accumulated. However, the Fig. 8 panel b showed that LCL161 treatment inhibits RIP1, TRADD, TRAF2 and cIAP complex assembly in cells in which the cIAP1&2 degradation is blocked by MG132 (as shown in the input). Thus, the release of RIP1 from the complex is not related to cIAP1&2 degradation. MG132 is a proteasome inhibitor, which inhibited the degradation of cIAP1&2, but it did not inhibit the ubiquitination of cIAP1&2. It is possibly because the ubiquitination of cIAP1&2 will lead to both degradation of cIAP1&2 and the release of RIP1 from the complex. Furthermore, we found that caspase-8 was activated through combining with RIP1 and FADD in the presence of LCL161 and paclitaxel. Additionally, we knocked down RIP1 with siRNA, revealing that RIP1 was indispensable in LCL161/paclitaxel-induced apoptosis. Therefore, we speculated that LCL161 decreased the self-degradation of the TRADD/TRAF2/RIP1/cIAP1,2 complex by accelerating the degradation of cIAP1 and cIAP2 and stabilizing RIP1 to form the caspase-8/RIP1/FADD complex, which then induced apoptosis by activating caspase-8 (Fig. 11).

At present, clinical trials of LCL161 and paclitaxel co-treatment are under way for patients with breast cancer [39]. It will be important to conduct an in vivo study to clarify the effects of the drug combination before clinical studies in lung cancer. In our present study, LCL161 and paclitaxel co-treatment prominently inhibited tumor growth without causing weight loss in mice. Thus, the therapeutic dose of the drug combination likely had few toxic effects on the mice. Furthermore, immunohistochemistry revealed that the drug combination degraded cIAP1 and cIAP2, activated caspase-3 and reduced the level of Ki67, findings that were in accordance with the results in vitro.

## Conclusions

In conclusion, co-treatment with LCL161 and paclitaxel may become an important way to improve the efficacy of paclitaxel, as well as to reduce the toxic effects of lung cancer treatment. However, more studies should be performed to further validate the function and mechanisms of the combination of LCL161 and other chemotherapeutics in different cancers, establishing a solid foundation for future clinical studies.

## Abbreviations

ATCC: American Type Culture Collection
BIR: Baculovirus IAP repeat
cIAP: Cellular inhibitor of apoptosis protein
FACS: Fluorescence-activated cell-sorting
FADD: FAS-associated death domain protein
IAP: Inhibitor of apoptosis
NF-kB: Nuclear factor kappaB
NIK: NF-kB inducing kinase
NSCLC: Non small cell lung cancer
PBS: Phosphate buffered saline
PI: Propidium iodide
RING: Really interesting new gene
RIP1: Receptor-interacting protein 1
Smac: Second mitochondria-derived activator of caspases
TNF: Tumour necrosis factor
TRADD: TNFR-associated death domain
TRAF2: TNF receptor associated factor 2
XIAP: X-linked inhibitor of apoptosis
zVAD.fmk: N- benzyloxycarbonyl-Val-Ala-Asp-fluoromethylketone
